# The Impact of Mood, Familiarity, Acceptability, Sensory Characteristics and Attitude on Consumers’ Emotional Responses to Chocolates †

**DOI:** 10.3390/foods11111621

**Published:** 2022-05-30

**Authors:** Annchen Mielmann, Neoline Le Roux, Innike Taljaard

**Affiliations:** The Africa Unit for Transdisciplinary Health Research (AUTHeR), School of Physiology, Nutrition and Consumer Sciences, North-West University, Potchefstroom 2531, South Africa; neoline.leroux@nwu.ac.za (N.L.R.); innike@gmail.com (I.T.)

**Keywords:** emotional response, sensory attributes, consumer behaviour, consumer variables, chocolate, structural equation model

## Abstract

Studies on emotions linked to sensory characteristics to understand consumers’ choice behaviour have grown in number rapidly. Internal consumer behaviour variables, namely mood, familiarity, acceptability, and attitude (MFAA), have been found to influence emotional response. The aim of this paper was to determine the impact of MFAA on consumers’ emotional responses towards chocolate as well as the effect of the sensory characteristics of chocolate on consumers’ emotional responses. Upon ethical approval, three chocolates were selected by a trained sensory panel based on 14 sensory attributes regarded relevant. Screened respondents (*n* = 149) completed an online survey based on the tasting of the chocolates by means of a home-use test (HUT). The questionnaire captured consumers’ mood (Quick mood scale), familiarity (QFFQ), acceptability (FACT), the sensory characteristics of the chocolate samples and emotional response (EsSense25 Profile), and lastly attitude (ACQ). Descriptive and inferential statistics were examined to answer the hypotheses of the study. The findings indicate that emotions are related to the bitter sensory attributes of chocolate and that this emotional response is influenced by MFAA variables, supporting the known fact that consumer behaviour is complex and multi-dimensional. Internal consumer behaviour variables play an important role in the emotions experienced during the consumption of chocolate. Investigating the relative importance of consumer behaviour components in sensory studies could allow for the design of food products such as chocolates based on a more “holistic” view of the consumer.

## 1. Introduction

Chocolate is consumed worldwide by consumers, and this continuously growing market is projected to have a value of USD 190 billion by 2026 [1]. Chocolate has the potential to arouse sensory pleasure as well as positive emotions due to its good taste [2], and its unique sensory properties, hedonic appeal, and psychoactive agents such as methylxanthines and cannabinoids that resemble drug-like effects on humans [3]. In addition, chocolate has been found to increase activity, reduce fatigue, and impact emotions by elevating mood and evoking joy and reducing depressive symptoms [4,5], while also influencing cognition, sensorial responses, and memory, whether positive of negative [6,7], and might even be good for human health, particularly cardiovascular health, if consumed in moderation [8,9]. Whilst chocolate is appealing for its hedonic properties, one’s social setting and/or culture may affect whether one chooses to consume it due to negative emotions, particularly that of guilt [10], which may be induced by cognitions based on culturally and societally determined attitudes to one’s health and body weight [11,12]. However, due to the strong emotional side of chocolate, affective experiences may be linked to chocolate consumption, both as precursors and consequences [4], as it has emotional benefits [13], and is even deemed an emotional experience in itself [14] and therefore has been the subject of various emotion studies and is thus considered a suitable product for an emo-sensory study.

The food industry is moving towards harnessing product-related emotions to obtain a competitive advantage [15], as negative emotions can be evoked within consumers when they are dissatisfied with new food products, which aligns with market performance [16]. Whilst overall-liking questions have traditionally been combined with the sensory characteristics of products in consumer tests to guide product design, some researchers have started tapping into the field of emotion measurement to uncover and assess these likes and dislikes on mostly a product or category level [17].

Research has highlighted the need for consumer-focused sensory research [18] and the consideration of behavioural observation methods. Unfortunately, limited studies have been conducted to investigate the relationship between consumer behaviour variables such as mood, familiarity, product acceptability, attitude, and emotional response to food products. Consequently, establishing possible relationships for an emotion-laden product such as chocolate can provide insights into the emotions that influence product acceptance and how these internal consumer behaviour variables influence consumers’ emotional responses to food products [19].

Accordingly, the present study aimed to determine and represent the relationships between consumer behaviour variables, and the sensory characteristics of chocolate, and emotional response. These relationships will add value to the discipline of food, sensory and consumer sciences, being first of a kind, since one of the main challenges for scientists is to provide actionable information to guide food product formulation design which will fit consumer demands or make specific changes to product formulations [20], and not only to provide product descriptions [21,22]. Moreover, determining relationships between consumer behaviour variables, sensory characteristics, and emotional responses could support the preference of a product formulation, in this case milk chocolate, which is fully aligned with consumer preferences [23], as this necessitates a good comprehension of consumers’ perception of food products [24], which could guide product development in the food industry [25,26,27].

## 2. Theoretical Background

**Figure 1:**
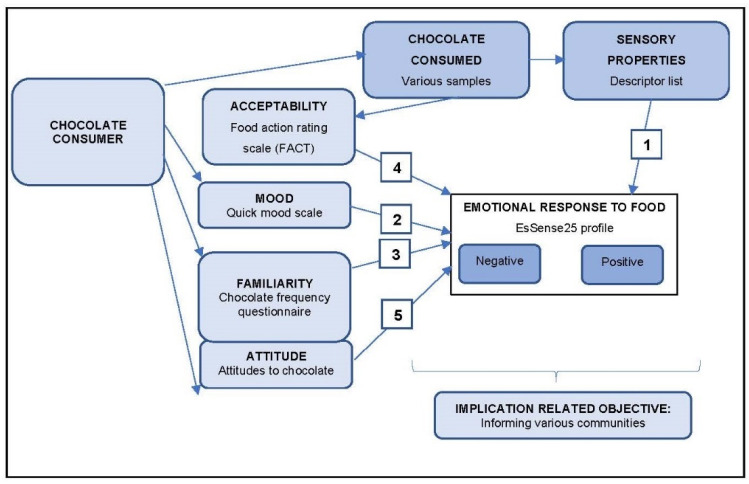
Framework of the study.

The framework in Figure 1 outlines the theoretical connection between the consumer behaviour variables mood, familiarity, acceptability and attitude, and the sensory properties of chocolate and emotional response.

### 2.1. Emotional Response

An emotional response is “a reaction to a particular intra-psychic feeling (an emotion), accompanied by physiological changes that may not be outwardly displayed, but motivate or bring about some action or behavioural response” [28]. An emotional response to a stimulus takes place in two stages—a primary sensory-motor response which takes place beneath conscious awareness, and a secondary process of awareness of the emotional experience [29]. Emotional expressive behaviour reflects the combined influence of the inner emotional impulse and factors governing the expression of certain emotions. There is consensus that emotions consist of three components: subjective experiences, physiological- and behavioural responses [30].

Food-evoked emotions are described as “a brief but intense physiological and/or mental reaction to a food or beverage item” [31]. Food-evoked emotions can influence or predict consumers’ food choice [32], as emotions provide an internal stimulus that elicits a beneficial or even corrective choice [33]. “Food-evoked emotions as motivators for food choice” have thus received considerable attention [34].

Emotional reactions to the consumption of foods play an important role in the acceptance of products in the market [19], and thus product-focused emotion research could provide a deeper understanding of these product experiences by consumers that leads to acceptance [35]. Consumers experience various emotions in their relationships with food products, and these emotions can be evoked by a variety of aspects such as sensory properties, functionality, usability, the social implications of using or owning the product, memories or associations attached to the product, or experience and learning [18], or the anticipated impact of product usage or ownership [36].

The connection between food and emotion is seen in the influence of emotion on food choice and consumption, and when consuming food, it can influence people’s moods and feelings, and thus the emotional response to food is not a stand-alone, one-stage process but rather a continuous loop.

### 2.2. Measuring Emotional Response

A common approach for the measurement of food-related emotion is the application of self-reported ratings of emotional terms in questionnaires such as the EsSense Profile [31] or its reduced version known as EsSense25 [37,38]. Several food-specific questionnaires have been developed, of which the Essense Profile appears to be the best validated and has gained influence in the field of sensory science [18]. It is considered as ‘the’ illustrative example of explicit measurement method [39]. The EsSense profile contains a relatively large number of emotional terms based on the finding that people tend to describe food products using a large number of terms [40], and this broad and detailed list of emotional terms delivers a more detailed emotional profile of a product which could have added value for marketing purposes [18]. Other than popularity, it has proven relevance across many product categories [18], including chocolate [26,27]. The EsSense Profile has, however, been criticised for containing too many emotional words associated with pleasure and too few words associated with displeasure [18]; however, the general idea that food elicits mainly positive emotions might explain the dominance of positive emotional terms in emotion lists [39]. Some emotion measurement methodologies and tools have, however, been stress-tested for various food products including chocolate, and the standardised tool such as the EsSense profile has also been applied to chocolate research studies with great success. In a study conducted by [41] which included chocolate as one of the products assessed, the emotion ratings obtained in repeated tests without evoked contexts were highly correlated and therefore the EsSense tool was deemed stable to measure emotional response to chocolate.

### 2.3. Sensory Characteristics

Sensory characteristics are a primary source of food-related emotion and are consequently considered an emotional feature. Visual, olfactory, and tactile characteristics of foods could have a direct emotional effect [36] and experience [42], seeing as the emotional profile of a product could be based on its intrinsic sensory characteristics [43]. This sensory perception, combined with consumer emotion, plays a significant role in building an emotional connection between consumers and products or brands [44]. Therefore, the provision of a sensory experience and the resultant emotions allow consumers to perceive, interpret and give meaning to individual experiences or to a food product or concept on a cognitive level.

### 2.4. Linking Emotions to Sensory Characteristics

When sensory characteristics, emotional experiences and cognitive responses are integrated, consumers form a view of what is happening around them [45]. Emotional profiling provides supplemental information alongside the sensory profile of food products, and therefore researchers attempt to obtain joint sensory and emotion profiles [39] to establish “holistic” views of products. These two concepts have even been combined into the hyphenated phrase sensory-emotion, referring to the sensory-emotion space or profile of a product [46].

This need for an improved conceptualization of individuals’ food experience led to the integration of emotional responses into consumer and sensory research [47] and the “application of consumer-based methodologies” during new food product development [48]. Although the food industry includes sensory affect and emotional conceptualisations in sensory testing, the theoretical and methodological supports are still premature [40]; however, new and more rapid methodologies are being developed in this field, with the aim of easy application and gaining fast responses [49].

It is noted that modern consumers seek emotional experiences and relationships with brands [50] and receive these from products via sensory perceptions [15]. Another perspective is that all objects have emotional features, and these features are observed by our sensory channels which are essential in affecting emotional responses. Therefore, sensory experiences evoke consumers’ emotions, as a sensory stimulus is an essential precursor of emotions and they are closely intertwined, affecting one another in both directions [51]. Various studies have investigated emotional response to food or beverage products, and the association between emotion and the specific sensory characteristics of foods are now being explored by many researchers, as sensory properties have been indicated as one of the sources of food emotion [52,53]. However, the development of methodologies to measure this link has been slow and quite varied, as although sensations and emotions are linked, how they are linked is a matter of debate amongst researchers. As consumers are not necessarily aware of sensory-induced emotions and may not be able to verbalise them [51], measuring explicit views is generally the approach used in emotion research [39] although it may not fully capture all the relevant results.

### 2.5. Consumer Behaviour

Another possible explanation for the link between food products and emotions is the role of consumer behaviour. Mood, familiarity, acceptability, and attitude are consumer behaviour variables that influence consumers’ experiences, preferences, favourability, and future use of food products [54]. Limited research has however investigated the association between the identified consumer behaviour variables, and the impact on consumers’ emotions. Consumer behaviour towards food is regarded as highly complex, with many external and internal influences [55], and thus the impact of emotional response on food choice and consumption [56], motivation to eat and amount of food consumed [57], acceptability, purchase intention and attitudes has been examined to a great extent [47]. Many studies have focused on the relationship between food and emotion, with only some incorporating or somewhat considering selected consumer behaviour variables. Food-related behaviour has been indicated to occur due to intuition, to take place on an emotional level, and be impacted by external and regulatory behaviours [58]. By measuring emotion, we may indirectly tap into the underlying motivations for food intake irrespective of this behaviour type, which clearly may not be related to only sensory properties [59].

### 2.6. Mood as a Consumer Variable

A positive mood evokes a positive emotional experience, whilst a negative mood evokes a negative emotional experience. Mood has been demonstrated to impact the emotional response, as the emotions evoked by a food stimulus are based on the individual’s internal state, and therefore the emotional response relies not only on the nutritional- or physical state but also on the current mood of the consumer [36]. A specific mood can also affect both the intensity and valence of a sensorily evoked emotional response [60]. Emotions thus seem to be dependent on consumers’ mood and psychological state, as those in a positive or negative emotional state have provided varying evaluations of food [57]. Based on the research findings, the present study proposes the following hypothesis:

**Hypothesis** **1** **(H1).** *Mood impacts emotional response.*

### 2.7. Familiarity as a Consumer Variable

Familiarity relates to a certain time during which a consumer has spent processing information about a product such as chocolate, regardless of the type of or extent of the processing. This processing of information creates knowledge of and attitudes towards products and brands which could influence products’ emotional and sensory profiles [61]. In addition, conceptual associations, e.g., the association between chocolate and happiness, are based on experience and learning from either internal sources, e.g., development of a positive mood when consuming a chocolate, or external experiences, e.g., linking the chocolate to positive memories or events such as Easter and Christmas [15]—consequently, some of these associations are emotional connotations [43], and therefore product familiarity is often studied for its influence on food-elicited emotion [57]. As a result, the possible effects of chocolate product familiarity on emotional response should be considered [26]. Considering the above, the following hypothesis is proposed:

**Hypothesis** **2** **(H2).** *Familiarity with chocolate impacts emotional response*.

### 2.8. Acceptability as a Consumer Variable

A high acceptability of food products evokes a positive emotional experience, whilst a low acceptability of food products evokes a negative emotional experience. Measures such as acceptability or liking, using The Food Action Rating Scale (FACT), are applied to understand consumer preference and food choice behaviour [62]. It is, however, crucial for sensory studies to move beyond measuring liking only by evaluating a broader range of insights into individuals’ involvement with food products [41], since food liking is complex and choice behaviour is influenced by many intrinsic (sensory) and extrinsic factors [37]. Researchers have emphasised the need for more reliable and differentiated measures of choice behaviour than liking or acceptability ratings, and additional studies are required to comprehend the association between emotion and the acceptability of foods and why this relationship varies, as various examples of highly acceptable products with different emotional intensities have been reported [31]. Emotions which are firmly linked to sensory experience could determine the preference for chocolate products [19], and combining sensory measures and emotions could increase the predictability for liking [63]. Based on these research findings, the following hypothesis is proposed:

**Hypothesis** **3** **(H3).** *Acceptability of chocolates impacts emotional response.*

### 2.9. Attitude as a Consumer Variable

While it is argued that emotions are an instinctive reaction towards sensory characteristics of food products based on associative learning, consumers tend to develop complex emotions towards foods [57] which influence their attitudes as well as behaviour and reactions. Consumers’ emotions towards food products make up the affective constituent of attitude, and these emotions are mainly interpretative in nature, as they determine how an object such as a food product makes one feel. These emotions thus compile consumers’ overall evaluation of food products as pleasant or unpleasant and are consequently often considered the fundamental component of an attitude. However, consumers’ attitudes towards food furthermore exist or change correspondingly based on conditioning, but they are also influenced by different emotional states [57]. Studies found that a negative emotional response is inversely related to attitude change, whereas a positive emotional response is positively related to attitude change [64].

Some studies on emotional responses to foods have included attitudinal measures as an indication of the consumer’s satisfaction with the product and as a predictor of the consumer’s future behaviour towards the product [43]. For example, a study conducted by [65] found that consumption frequency and attitudes towards chocolate did have an influence on the emotional profiles. In addition, a study by [27] confirmed that emotional satisfaction was the main reason for chocolate consumption. Considering the aforementioned findings, the following hypothesis is proposed:

**Hypothesis** **4** **(H4).** *Attitude towards chocolates impacts emotional response.*

### 2.10. Sensory Properties and Emotional Response

Researchers who studied chocolate as a subject stated that sensory characteristics acted as a motive for emotional response and found a link between specific sensory properties and emotional conceptualisations [39]. These conceptualisations pointed out that flavours of food products are associated with a wide spectrum of feelings—for example, a creamy texture and sweet taste is associated with being fun, easy-going and comforting. Studies also indicate that emotion plays an important role in acceptability [15]. It is therefore argued that if emotion influences acceptability and sensory properties as well, the sensory properties of a product will in fact impact the emotional response of consumers on a food product with regard to how they will evaluate and experience a food product [26]. Based on this research, the following hypothesis is proposed:

**Hypothesis** **5** **(H5).** *Sensory properties of chocolate impact emotional response.*

## 3. Materials and Methods

**Figure 2:**
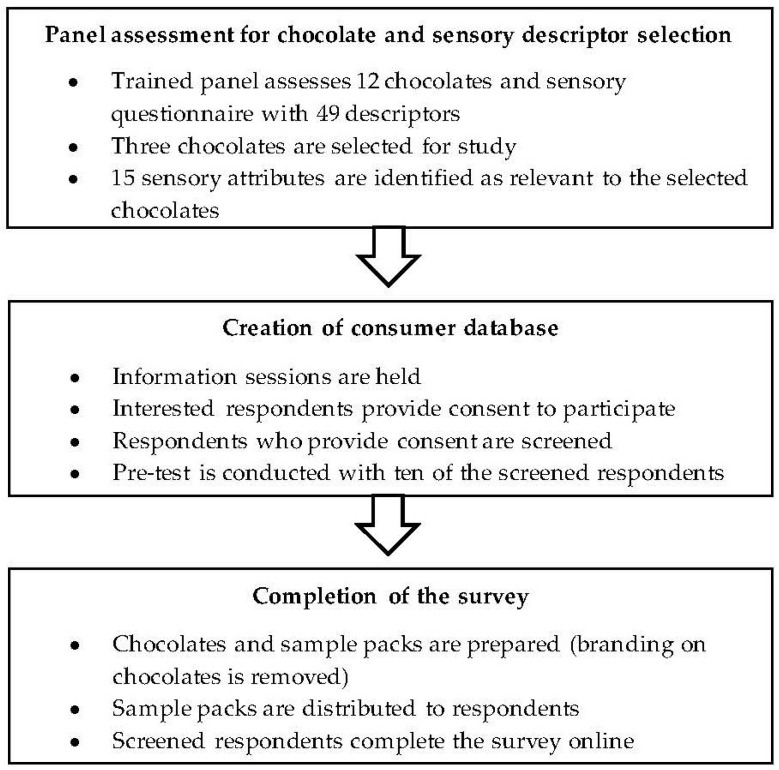
Main steps of the study.

The researchers obtained ethical approval from the Health Research Ethics Committee (HREC) of the Faculty of Health Sciences of the North-West University of the Faculty of Health Sciences (Approval number NWU-00029-17-A1) for research projects with human participants. The main steps of the study are provided in Figure 2.

### 3.1. Selection of Sensory Characteristics and Chocolate Samples

To ensure that variability in chocolate types did not influence respondents and impact results, only milk chocolate in tablet format was selected for the study, as it is the most popular format in South Africa. Consumers also prefer plain milk chocolate in general over dark chocolate [66]. As a consumer-centric study, the aim was to include real products to capture real responses to products, and thus no model foods were included.

A trained panel employed by a well-known Swiss fragrance and taste company in South Africa was used to refine the list of sensory characteristics and identify the chocolates to be included in the consumer study. This trained panel consisted of six panellists who had all been trained to assess and measure the intensity of the sensory characteristics of chocolate. They had extensive exposure across multiple food product categories, and they received specific training on the chocolate category and, more specifically, on the products available in the South African retail sector [67].

For the sensory characteristics, the researchers in [48] stated that sensory terms could be selected for studies by reviewing results from previous qualitative or quantitative consumer studies, and this approach was applied for this study. As a starting point, a list of 49 sensory descriptors for chocolate was compiled by reviewing the sensory descriptor lists generated in 13 different studies focusing on chocolate. For this study, 12 milk chocolates available in the South African retail space were used. The trained panel reduced the sensory list to 14 characteristics (Appendix A, Section 4) which best described the 12 chocolate samples and the variances between the samples in the market, which is also a number which is more manageable with a consumer panel. Those sensory characteristics that were not applicable to the 12 chocolates evaluated by the trained panel were therefore eliminated for the preparation of the final sensory descriptor list for the questionnaire (Appendix A, Section 4).

The trained panel was then employed to reduce the amount of chocolate samples. The list of 14 sensory characteristics compiled for the consumer questionnaire (Appendix A, Section 4) was then used by the trained panel during which three different milk chocolates were selected from the set of 12 chosen chocolate products as they were found to be sensorily varied by the trained panel. As the trained panel found the chocolates to be varied for all sensory attributes measured when the products were evaluated with the same questionnaire that consumers would be using, it was expected that untrained consumers would also be able to identify these variations and rate the intensity of the sensory attributes accordingly. Connecting the data from a trained panel to a consumer test for a common set of products is a very powerful development tool [68].

Trained panellists performed the evaluation independently in individual sensory evaluation booths at a sensory evaluation facility in Johannesburg. They consumed bottled mineral water (chocolate 3 brand) and water biscuits (Carrs brand) as palette cleansers before commencing evaluations. The three chocolate products which were thus included were an artificially sweetened milk chocolate aimed at diabetics (chocolate 1); a premium imported chocolate positioned towards indulgence (chocolate 2); and a mainstream chocolate marketed as an everyday luxury (chocolate 3). As no data were available on the emotional response to chocolate in South Africa, it was not possible to evaluate these products to select emotional terms which would describe and differentiate between the samples.

### 3.2. Chocolate Sample Presentation

As the aim of this study was to measure the emotional response to sensory characteristics (intrinsic), samples were presented blind, unbranded, and unpackaged. Branding was removed by melting the surface of the chocolates with a hot spoon without distorting the surface or shape significantly, and the samples were then cooled down to room temperature (approximately 20 °C).

Sample preparation took place in a food laboratory where four blocks (20 g) of milk chocolate (3 chocolate samples) were then packaged in individual small zip-lock plastic bags (4 cm × 4 cm) and labelled with a three-digit code. Three chocolate samples were packed in a cooler bag to ensure they remained within room temperature range (15–25 °C) until consumed. This was repeated for all three samples sequentially. A combination of water and water biscuits was provided to respondents to cleanse the palate between chocolate samples. Each respondent was therefore provided with a cooler bag (sample pack) containing:

(1) the three chocolate samples;
(2) a 330 mL bottle of still mineral water;
(3) three Carrs water biscuits (weighing 8 g per biscuit, thus 24 g of biscuits was provided), sealed in a zip-lock bag, one to be consumed before the first, second and third chocolate sample was assessed.
(4) storage instructions (as to not compromise sample quality and integrity); and
(5) a reminder of the questionnaire instructions (such as the use of palate cleansers and the request to complete the questionnaire in one sitting).

### 3.3. Questionnaire

The questionnaire (Appendix A) was compiled on the SurveyMonkey website and consisted of six sections. The first section captured respondents’ mood using the Quick Mood Scale [69] that consisted of twelve adjectives which were the positive and negative representatives of six dimensions, namely friendly and aggressive, relaxed and anxious, clearheaded and confused, cheerful and depressed, well-coordinated and clumsy, and wide awake and drowsy. A 5-point unipolar verbal scale (0 = not at all; 1 = slightly; 2 = moderately; 3 = very; 4 = extremely) was implemented.

The second section measured respondents’ familiarity. The scale of the QFFQ [70] was applied with two variations: firstly, an option for “never” was included to record usage within specific sub-segments of the chocolate category. Secondly, the “seldom” section was labelled as “once a year/rarely”. A 5-point Likert scale (1 = daily; 2 = weekly; 3 = monthly; 4 = once a year/rarely; 5 = never) was implemented.

The third section determined respondents’ acceptability of the three chocolate samples. This was the first section where respondents were required to taste the chocolate samples. The FACT was utilised without any modifications to capture consumers’ perceived product acceptability, as it is a behaviourally or action-oriented approach to the scaling of food acceptability [71]. It was successfully applied [72], and reliability tests using the scale showed that the scale yields highly reliable results. It included nine statements (Appendix B, Table A1, Section 3) which represent different behavioural options regarding the frequency of consumption as well as motivationally related statements. The questions were presented in a multiple-choice format, as the respondents were instructed to only choose one option.

The fourth section captured the sensory characteristics of the chocolate samples. Responses with regard to the chocolates’ appearance (gloss), aroma (sweet), taste (bitter, burnt, cocoa powder, coffee, milky, sweet, vanilla), mouthfeel (adherence, creaminess, melting, mouth coating) and aftertaste (bitter) were recorded using the Rate-All-That-Apply (RATA) scale (0 = not at all; 1 = slightly; 2 = moderately; 3 = very; 4 = extremely) [22]. RATA is focused on intensity and is useful for in-depth characterisation [40], and is thus a more suitable technique in certain instances compared to Check-All-That-Apply (CATA), especially when the intensity of terms is the main difference [35], as these questions make it possible to obtain reliable scores for the intensity of sensory properties. As respondents were asked not only to select but also rate all the words from a list that apply to the product being evaluated, RATA is likely to trigger a more analytical mindset in respondents [73], it improves hedonic discrimination between samples, and opens up more opportunities for statistical analysis [22]. Furthermore, RATA questions have the advantage of using a similar approach to CATA questions, and therefore also enable quick and easy data collection from large consumer samples [73].

After the first chocolate was tasted, the questions on the sensory characteristics were completed, and the emotional response questions were then completed afterwards for the same product. This was then repeated for chocolate samples two and three.

The fifth section measured respondents’ emotional response. Consumers’ emotions were recorded after the sensory characteristics, as the emotional response to food is often based on the sensory qualities of a single taste [56], and intrinsic sensory properties have a strong association with emotions [74]. The EsSense25 Profile [38] was implemented and contained positive emotions (active, adventurous, calm, enthusiastic, free, good, good-natured, happy, interested, joyful, loving, nostalgic, pleasant, satisfied, secure and warm), negative emotions (bored, disgusted, worried), and uncategorised emotions (aggressive, guilty, mild, tame, understanding, wild). The RATA scale (0 = not at all; 1 = slightly; 2 = moderately; 3 = very; 4 = extremely) was implemented. The scale recommended for use along with the EsSense profile [38] is a 5-point rating scale. However, as per the recommendations of previous studies [15], when the same or similar scales were used to measure emotion and sensory properties, the RATA approach was used for the sensory and emotion measurement sections in the questionnaire as well as to measure the intensity of the emotional response.

In the last section, attitude (negative, emotional, functional, obsession) was measured by using the Attitudes to Chocolate Questionnaire (ACQ) that included 24 statements without any changes or modifications [75]. A 5-point Likert scale (1 = strongly agree; 2 = agree; 3 = neither agree nor disagree; 4 = disagree; 5 = strongly disagree) was implemented.

The questionnaire was developed in English, which is a language generally understood and spoken by most South Africans [76] and is the most dominant language used for research in South Africa [77]. The questionnaire in this study was applied as computer-assisted self-interviewing (CASI), designed on the SurveyMonkey website. A comparison of the results of a pen-and-paper personal interview (PAPI) with CASI found that CASI had more absolute item response rates than the pen and paper method [78], which would benefit this study where full completion of each section was required for correlation analysis. It also saved significant time, improved data accuracy due to the length of the questionnaire of this study and reduced respondent fatigue by allowing consumers to complete the survey at their own pace and allowing them to take breaks as they needed. It offered the flexibility to record responses in the respondents’ natural setting, which is important for emotional response studies, and thus created an evoked chocolate consumption context. Self-administered questionnaires are also best for accurately capturing honest emotional responses as well as answers to attitudinal questions, as health problems of a sensitive nature are often under-reported in telephone or face-to-face interviews compared with self-administered surveys [79].

### 3.4. Data Collection

Respondents were requested to collect their sample packs from a central location in Johannesburg from November to December 2017. After the sample packs had been collected (within 24 h after collection), respondents received an email with a link to the questionnaire on the SurveyMonkey website, with a clear indication of the time frame within which it needed to be completed. They were able to complete the online survey at a time and place that suited them best within 72 h to protect them from any possible discomfort, stress, or fatigue. Respondents were also able to take comfort breaks if needed to reduce fatigue due to the length of the questionnaire. Discomfort stress can occur when respondents feel that they are under pressure to complete the online questionnaire during workhours, which may have led to emotional discomfort and could in fact have influenced the emotional responses received. For the actual tasting of the chocolates, respondents were instructed in the questionnaire to taste all three chocolate samples before completing the questions on acceptability, sensory characteristics, emotional response, and attitude.

### 3.5. Research Setting

The actual research setting was when and where respondents completed the online questionnaire. The objective was to study consumer’s everyday consumption experience in a natural context, and subsequently, the researchers did not want to remove the respondents from their normal, everyday situation but rather aimed to collect the data in a setting with as little disruption of the typical setting as possible [80], and therefore not in a sensory laboratory. To achieve this, respondents were requested to complete the tasting where they usually consumed chocolate to mimic the “natural” setting where consumption usually took place. Therefore, this study used a home-use test (HUT) where the respondents could test the chocolate samples with more time in their familiar home environment [81]. With HUT, the products were evaluated in the consumers’ normal environment and over a period, and the evaluation was based on stabilised use rather than a first impression [82]. It is generally assumed that a more realistic HUT yields more relevant hedonic data than central location tests despite the uncontrolled conditions, because in HUT, the products are evaluated in the consumers’ normal environment and over a period, the evaluation is based on a stabilised use rather than a first impression [81].

### 3.6. Respondents

During November 2017, advertisement posters were placed on notice boards on the campus of the WITS University’s Business School in Johannesburg, South Africa to inform potential respondents that the study would take place, and should they be interested in participating, respondents could attend one of six scheduled information sessions. During the information session, aspects such as the objectives of the study, the recruitment criteria, expectations of the respondents, the ingredient list of the chocolates for the identification of allergies, and ethical considerations were discussed. All interested respondents received an e-mail containing an electronic link to the screening questionnaire on the SurveyMonkey (https://www.surveymonkey.com/) (accessed on 13 November 2017) (San Mateo, CA, USA) website. Respondents had to provide consent electronically to participate in the research study.

The sample population included 149 South African adult consumers who live in the Gauteng Province within the Johannesburg municipal district. Conducting a study in this district also meant that it would be possible to find consumers who consume chocolate frequently, and from a retail perspective, consumers from this area would also have easy access to a wide variety of chocolate products due to the ongoing large expansion of supermarkets in this urban environment of South Africa. Respondents (18 years or older) should have consumed chocolate at least once in the past year to have a level of familiarity with the chocolate category to be considered a chocolate consumer.

Table 1 presents the demographic profile of the respondents. The respondents consisted of 26% males and 74% females, all older than 18 years. Most of the respondents were between the ages of 30 to 39 years (37%). Respondents’ educational level ranged from grade 12 (high school) to a post-graduate degree, with most of the respondents being in the possession of a diploma/certificate (33%) and a post-graduate degree (33%).

### 3.7. Data Analysis

The statistical software IBM^®^ Statistical Package for the Social Sciences (SPSS^®^) version 25 (www.ibm.com/products/spss-statistics) (Chicago, IL, USA) was implemented. For the demographic profile, descriptive statistics were applied. Cronbach’s alpha was calculated to assess the internal consistency and reliability of the scale items of the questionnaire.

An exploratory factor analysis of the results was performed using the principal component method of extraction for the dataset to reduce and group the number of variables and, to create pattern matrixes for the variables. An Oblimin rotation was performed since factors were expected to be correlated. The factor analysis for the sensory and emotional attributes was performed on the three chocolates included in the study individually to determine whether the data yielded the same factors for all three chocolate samples. This resulted in the extraction of two factors for sensory characteristics, namely sweet and bitter, and two for emotions, namely positive and negative. For the consumer behaviour variables, factor analysis yielded two factors for mood, namely positive and negative, two for familiarity, namely small chocolates and large chocolates, and four for attitude, namely negative, functional, emotional and obsession. The internal consistency of all factors was high, with acceptable Cronbach’s alpha scores for all factors (α ≥ 0.6) [83].

To investigate the relationship and thus the possibility of correlations between mood, familiarity, acceptability and attitude and emotional response to the three chocolates, Spearman’s rank order correlation coefficients were calculated (r). Interpretation of the Spearman’s rank order coefficients was based on [84], where r = 0–r < 0.3 is viewed as small, r ≥ 0.3–r < 0.5 as moderate, and r ≥ 0.5 as strong. r ≥ 0.3 was viewed as a tendency to correlate.

Multiple regression analysis was implemented to determine the relationship between the sensory characteristics and consumers’ emotional response, as this analysis can predict the outcome of a dependent variable—in this case, emotional response based on the interaction with explanatory or independent variables, namely the sensory characteristics of chocolates. A separate multiple regression analysis was performed for each single chocolate with only one of the emotion factors (either positive or negative) along with the two sensory factors (both bitter and sweet).

Face validity was achieved by consulting the statistical consultation services of the university to assess the analysability of the questionnaires and data they would yield. Confirmatory factor analysis was performed to ensure construct validity of each of the scales of each section of the questionnaire, to determine whether the variables being investigated are interrelated or not and to measure how valid the instruments were in testing the different constructs. To ensure content validity, qualified personnel within the department of consumer science who have backgrounds regarding the research topic were consulted. For reliability, a pre-test was conducted to ensure that the questionnaire was a reliable instrument, and this was deemed necessary as the questionnaire was self-administered and was presented as CASI.

## 4. Results

### 4.1. Mean Scores

The mean values for the acceptability and emotional responses for the three chocolate samples are provided in Appendix B, and only significant findings are reported. The premium chocolate (sample 2) was found to be significantly (*p* ≤ 0.05) more acceptable than the diabetic chocolate (sample 1) and the mainstream chocolate (sample 3), which achieved similar acceptability mean scores. While the diabetic chocolate achieved a significantly (*p* ≤ 0.05) better acceptability mean rating than the other two chocolate samples, it appears that consumers did not experience significantly (*p* ≤ 0.05) more positive emotion during the consumption of the diabetic chocolate. The relationship between negative emotion and the acceptability of the diabetic chocolate sample was stronger than the positive emotion relationship. The emotional response to these chocolates was thus not reflected by the mean acceptability ratings.

Table 2 presents the mean scores of the different constructs. The mean score for acceptability was the highest (4.23), followed by emotional attitude (3.01). The mean scores for positive mood and emotion were higher than for negative mood and emotion. Respondents were slightly more familiar with larger chocolates and respondents’ mean score for sensory sweetness was higher than sensory bitterness.

### 4.2. Factor Loadings, p-Values and Cronbach’s Alpha

Table 2 shows the factor loadings, *p*-values, and Cronbach’s alpha (α) for mood, familiarity, acceptability, attitude, sensory characteristics, and emotional response. The factor loading of the constructs exceeded the minimum cut off point of 0.50, and therefore all items were included for the interpretation of the consumer variables on respondents’ emotional response, and therefore convergent validity was confirmed. Cronbach’s alpha for the identified constructs ranged from 0.717 to 0.774, which exceeded the minimum acceptable value of 0.70 and revealed good internal consistency of the scale items in the questionnaire.

### 4.3. Correlations

Spearman’s rank order correlations (r) were also determined to describe the relationship between the consumer variables, mood, familiarity, acceptability and attitude and emotional response for the three chocolate samples, as presented in Table 3.

#### 4.3.1. Mood

Only three significant (*p* ≤ 0.05) positive relationships were noted, two with small r values (r = 0–r < 0.3), namely between positive mood and positive emotion for chocolate 3 (r = 0.245, *p* ≤ 0.05) and negative mood and guilty emotion, also for chocolate 3 (r = 0.218, *p* ≤ 0.05), and one moderate tendency to correlate (r ≥ 0.3- < 0.5) between the negative mood and guilty emotion for chocolate 2 (r = 0.335, *p* ≤ 0.05).

#### 4.3.2. Familiarity

Significant (*p* ≤ 0.05) positive correlations were identified between the positive emotion factor and both the small format (r = 0.209, *p* ≤ 0.05) and large format (r = 0.232, *p* ≤ 0.01) chocolate factor during the consumption of chocolate 2, as well as between the positive emotion factor and both the small format (r = 0.272, *p* ≤ 0.01) and large format (r = 0.2321, *p* ≤ 0.01) chocolate factor during the consumption of chocolate 3.

#### 4.3.3. Acceptability

All correlation (r) values between acceptability and emotion for chocolate 2 and chocolate 3 were small (r = 0–r < 0.3). For chocolate 1, however, both positive and negative emotion had a moderate (r ≥ 0.3–r < 0.5) significant (*p* ≤ 0.05) correlation with acceptability, with positive emotion positively correlated with acceptability (r = 0.463; *p* ≤ 0.01), while negative emotion negatively correlated with acceptability (r = −0.460; *p* ≤ 0.01). This indicates that the emotional response to chocolate 1 was more accurately captured by its mean acceptability than the other two chocolates. The mean factor score for the positive emotion factor for all three chocolates was, however, very similar (chocolate 1 = 0.780; chocolate 2 = 0.802; chocolate 3 = 0.795), while a larger differentiation was noted for the mean factor score for the negative emotion factor (chocolate 1 = 0.593; chocolate 2 = 0.609; chocolate 3 = 0.525). Despite this, chocolate 2 was found to be significantly (*p* ≤ 0.05) more acceptable than chocolate 3 and chocolate 1, which achieved similar acceptability mean scores. While chocolate 2 achieved a significantly (*p* ≤ 0.05) better acceptability mean rating than chocolate 3 and chocolate 1, it appears that consumers did not experience significantly (*p* ≤ 0.05) more positive emotion during the consumption of chocolate 2. The relationship between negative emotion and acceptability of chocolate 2 was stronger than the positive emotion relationship. The emotional response to these chocolates was thus not reflected by the mean acceptability ratings.

#### 4.3.4. Attitude

Only one moderate (r ≥ 0.3–r < 0.5) significant (*p* ≤ 0.01) positive correlation was noted between guilty emotion experienced during the consumption of chocolate 3 and a negative category attitude (r = 0.309, *p* ≤ 0.01). A functional attitude to the category had very low Spearman’s rank order (r) correlation values with any of the emotion factors for all the chocolates. The correlations between the functional attitude factor and the positive emotion factors were positive for all three chocolates, while the correlations between the functional attitude factor and the negative emotion factor were negative for chocolate 2 and chocolate 3 but positive for chocolate 1, indicating that those consumers who had a functional approach to the category experienced more negative emotion during the consumption of chocolate 1. Consumers with a functional approach were also more likely to experience guilt during the consumption of chocolate 2. Generally, the functional attitude factor had lower Spearman’s rank order (r) correlation values with emotion than the other attitude factors, indicating that functional chocolate consumers may experience less emotion than those of the other attitude factors.

A negative attitude resulted in a negative correlation with positive emotion for all three chocolates, and a positive correlation with negative emotion, and thus these consumers were less inclined to experience positive emotion and more inclined to experience negative emotion during chocolate consumption. A respondent with a negative attitude is also more likely to experience guilt when consuming chocolate, as the negative factor was significantly correlated (*p* ≤ 0.01) with guilt for all three chocolates (chocolate 1r = 0.230, *p* ≤ 0.01, chocolate 2r = 0.299, *p* ≤ 0.01, and chocolate 3r = 0.309, *p* ≤ 0.01). Guilt was thus experienced by this group of respondents irrespective of the chocolate type consumed and was thus possibly related to the category and not to the individual chocolates.

Only two significant correlations (*p* ≤ 0.05) were noted for the emotional and obsession attitude factor with the positive or negative emotion factors, both during the consumption of chocolate 3. The Spearman’s rank order correlation values (r) of these two relationships were, however, small (r = 0–r < 0.3). Furthermore, for chocolate 3, both factors correlated positively with guilt and negatively with negative attitude. The same was noted for chocolate 2, with positive emotions also positively correlating to these factors. For chocolate 1, however, while positive emotion correlated positively with both factors, so did negative emotion, while guilt had a positive correlation with emotional attitude and a negative correlation with obsession attitude. Chocolate 1 had lower correlation coefficients than chocolate 2 for positive emotions, and higher correlation coefficients than chocolate 2 for negative emotions, with very low guilt correlation coefficients. The highest guilt correlation coefficient was noted for chocolate 2. Thus, directionally, the diabetic chocolate evoked less emotion in these consumer types and chocolate 2 evoked more, both positive and negative, as well as guilt.

### 4.4. Regression

Table 4 indicates the results from this multiple regression analysis, and the R^2^, R^2^ adjusted, β-coefficients and *p*-values are reported for the sensory characteristics and emotional response.

The results indicate that for chocolate 1, only 9.5% of the variance of the positive emotion factor is explained by the sensory factors, while the sensory factors explain 24.1% of the variance of the negative emotion factor. The sweet sensory factor made a unique statistically significant positive contribution (β = 0.481; *p* ≤ 0.05) to the positive emotion factor, and a unique negative contribution to the negative emotion factor (β = −0.378; *p* ≤ 0.05), while the bitter sensory factor made a unique positive contribution to the negative emotion factor (β = 0.330; *p* ≤ 0.05), and no contribution to the positive emotion factor. Only the contribution of the sweet sensory factor to the positive emotion factor is viewed as practically significant (β ≥ 0.4).

For chocolate 2, only 6.4% of the variance of the positive emotion factor is explained by the sensory factors, while 0.0% of the variance of the negative emotion factor is explained by sensory factors. Only the bitter sensory factor made a unique statistically significant positive contribution (β = 0.427; *p* ≤ 0.05) to positive emotion. For chocolate 3, only 1.3% of the variance of the positive emotion factor is explained by the sensory factors, while sensory factors explain 2.0% of the variance of the negative emotion factor. Neither of the sensory factors made a unique contribution to either the positive or negative emotion factors.

The above results indicate that the variation in the percentage of the emotion factors, which is explained by the sensory factors, is based on the unique profile of the chocolates. As the three chocolates were found to be sensorily different, this resulted in the sensory attributes of chocolate 1 having a large (24.1%) influence on negative emotion and a smaller influence on positive emotion (9.5%), while the sensory attributes of chocolate 2 had a small (6.4%) influence on positive emotion only, and for chocolate 3 the sensory attributes had a negligible influence on both positive and negative emotion.

## 5. Discussion

This study determined the impact of mood, familiarity, acceptability, and attitude (consumer variables) on consumers’ emotional responses as well as the effect of the sensory characteristics of chocolate on consumers’ emotional responses.

A positive mood impacted a positive emotion (H1). Consumers enjoy eating hedonic foods particularly when in a positive mood. For example, researchers found that motivation to eat has been found to be higher during joy than during sadness and chocolate was rated as more pleasurable during positive emotions. Consequently, when in a positive mood, exposure to tempting food may increase the pleasure of eating and result in more intake [85].

Respondents’ familiarity with chocolates impacted a positive emotion. This confirms hypothesis 2, stating that familiarity impacts emotional response. Familiarity has an important connection to consumer experience [86], as it creates knowledge of products and brands which could influence a products’ sensory profile [87]. Studies confirmed that respondents consume familiar foods to relieve feelings of distress and anxiety and that novel foods cannot fulfil this need because they tend to evoke more feelings of anxiety. Furthermore, familiar foods such as chocolate represent the sense of perceived “comfort” and optimism [88]. Therefore, it is likely that the more familiar a consumer is with a certain food product, the more they can connect certain emotional responses to the product.

For acceptability, the acceptance of chocolates impacted a positive emotion, confirming the third hypothesis (H3). This might suggest that a sweeter or less bitter product is more likely to influence acceptability, which in turn affected positive emotion. Researchers investigated consumers’ emotions towards chocolate and found that consumers reported having mostly positive emotions when consuming chocolate [26]. Others studied the effects of emotional responses to certain foods on the prediction of consumer acceptance and agreed with the relationship found between the acceptability of food products and the emotional response [19]. A high correlation between liking and happiness exists where the association between the acceptance of products and emotional reactions occurred during the consumption of different food [19]. Sweetness presents a high negative correlation with bitterness, and chocolate samples with a higher intensity of bitterness obtained lower sweetness ratings [89]. Respondents in sensory research studies have, however, confirmed that they found the balance between the sweet and bitter taste to be of importance [90], and thus the extent of sweetness or bitterness may not be as relevant as the overall sensory experience based on this relationship. Thus, both the sweet and bitter sensory variables are equally important in influencing consumers’ acceptability of food products such as chocolate, as used in this study.

For attitude (H4), the emotion guilt was mostly experienced by consumers irrespective of the chocolate sample consumed, confirming that attitude towards chocolate impacts emotional response. Guilty should be specifically mentioned as an emotional attribute which is impacted by product type, as [31] found that guilt was most intense for chocolate, pizza, and fried chicken, while being least intense for mashed potatoes. It is thus concluded that attitude, as a complex consumer variable, impacts one’s emotional response towards food products, and that attitude can vary depending on the product type and how it makes one feel.

Between the two sensory factors, only sensory bitterness influenced negative emotion, while sensory sweetness did not affect either positive or negative emotion (H5). Researchers have been able to confirm a definitive relationship that exists between consumers’ bitter taste perception, emotions, and behaviour. As emotion is a component of a larger group of reactions known as affective feelings which also includes moods [40], and specific emotions present themselves as moods [67]; it can be concluded that mood could have influenced the experience of the bitter sensory variable and in turn emotion. Consumers that are sensitive towards bitter tastes tend to display negative emotions and emotional responses towards food and beverages with a bitter compound [91]. Moreover, it has been shown that when consumers are stressed, they tend to consume more sweet, high-fat products, as negative emotions can often be measured by eating sweet foods [2].

Researchers measured food-elicited emotions using facial expression recognition software and results showed that milk chocolate had the highest intensity of emotion “happy” and reduced the level of the neutral state [19]. The researchers investigated the influence of sensory attributes on consumers’ emotions and hedonic liking of chocolate and found that the premium-brand chocolate was associated with the highest number of positive emotions, whereas the traditional brand was associated with most of the negative emotions (“bored”, “disgusted” and “worried”). The drivers of liking were mainly positive and unclassified emotions [26]. Therefore, the consumption of chocolate products can intensify the manifestation of emotions.

### 5.1. Value of the Study

No studies are known to have been conducted to measure the impact of consumer behaviour variables on the emotional response to the sensory characteristics of chocolates, merging the field of sensory and consumer sciences to provide actionable product design insights. The findings of this study indicate that emotions are related to the bitter sensory attributes of chocolate, and that this emotional response is influenced by various internal consumer behaviour variables, which support the known fact that consumer behaviour is complex and multi-dimensional. These relationships have not previously been explored in conjunction; the various sub-relationships, specifically the relationship between emotional response and consumer behaviour in the context of sensory characteristics and between the consumer behaviour variables, have enjoyed very limited attention in the literature. It is therefore essential to understand the field of consumer behaviour from the viewpoint of multiple disciplines, as this would provide an opportunity to investigate and explain a phenomenon considering multiple disciplines. The results thus indicate how important it is to consider consumer behaviour aspects when sensory research is conducted, which would provide a new outlook on the execution and approach to consumer-focused studies.

Linking the sensory characteristics of chocolates and consumer behaviour, as presented in this article, can provide valuable information for the prediction of consumer behaviour. Furthermore, it is necessary to consider the importance of sensory components for product categories, going beyond correlating sensory attributes to liking. It is crucial to identify interrelationships between attributes involved [25]. From a new product development perspective, the selection of a food product formulation that is aligned to the furthest extent with consumer preferences is very important for the food industry, which requires a solid comprehension of consumers’ perception of products, both from a hedonic and sensory perspective.

From a consumer perspective, this study highlighted the importance of consumer behaviour variables in sensory research and could allow for the design of products such as chocolates based on a more “holistic” view of the consumer. Broadly, it brought the fields of sensory and consumer science together. Although various theories exist in these different disciplines which are well researched and defined, the combination and integration of these topics provide a different and unique point of view to what has been made known to date and that has also allowed for new insights to emerge; this combination is often overlooked or deliberately ignored.

### 5.2. Limitations and Recommendations

This research was based on self-reported consumption behaviours, and thus data related to familiarity may not be valid. It is also based on data collected from a relatively small sample, and therefore cannot be generalised to the entire South African population. Most respondents were female, and this could skew the results, as differences have been found between males and females in terms of emotional response and other variables investigated. A higher number of respondents had a tertiary education and were therefore more likely to be able to understand and respond to emotional terms, whilst this may not be true for a less educated population. Should anyone be interested in replicating this study, the addition of a new dataset generated amongst consumers recruited and interviewed in the same manner could offer the opportunity to make the findings even more generalisable. Furthermore, the study could be replicated with different consumer groups with changes to the data collection procedure to also validate the generalisability of the methodology that was implemented. In addition, considering that South Africa is regarded as a developing country, similar studies performed in developed countries may be valuable to further explore this research topic. The application of the methodology, tools and scales to collect data on other product categories would be valuable to explore beyond the chocolate category and to understand if similar relationships between emotion and sensory attributes and emotion and consumer behaviour exist beyond the chocolate sector.

Further studies, from a data analysis viewpoint, should, however, focus on a different approach to the analysis to uncover relationships, as the findings from the various correlation analyses performed in this study point to the possibility that underlying relationships within the set of sensory attributes and/or the set of emotional attributes may have influenced the overall association between sensory and emotional attributes. This may extend to the consumer behaviour variables which were investigated. Furthermore, the inclusion of demographic variables, consumers’ expectations and purchase intent, which are important from a South African perspective, should be considered in data analysis, as they could uncover significant relationships which were not considered in the current study, as the aim of this study was to include a homogenous sample size based on specifically identified inclusion criteria, rather than investigating the impact of demographics and other consumer behaviour variables as a further dimension of data analysis.

Lastly, the inclusion of a more extensive list of emotion and sensory attributes to better describe and differentiate samples and allow for the identification of unique relationships within chocolate samples, as well as universal relationships between samples, should be considered. Chocolate products could be more differentiated with a larger number of emotional responses, as consumers use a large quantity of emotional terms to assess food products [19], which is also true for sensory attributes. This could also extend to the generation of a chocolate-specific lexicon by South African consumers vs. the application of an internationally validated tool.

## 6. Conclusions

This study aimed to determine the impact of mood, familiarity, acceptability, and attitude (consumer variables) on consumers’ emotional response as well as the effect of the sensory characteristics of chocolate on consumers’ emotional response. The results confirm that an association exists between the sensory characteristics of chocolates, emotional response, and consumer behaviour variables. This study addressed the gap in the literature about consumer behaviour in relation to emotional response to determine what role internal consumer behaviour variables play in the emotions experienced during the consumption of chocolate. The study generated the first data within the field of emo-sensory research in South Africa and has shown that integrated studies are viable and valuable. Investigating the relative importance of consumer behaviour components in sensory studies could allow for the design of products based on a more “holistic” view of the consumer.

## Figures and Tables

**Table 1:** Descriptive demographic profile (*n* = 149).

Demographic Profile	Percentage of Respondents (%)	Number of Respondents (*n*)
Gender		
Male	26	38
Female	74	111
Age in years		
18–29	23	34
30–39	37	55
40–49	26	38
50–59	9	14
>60	5	8
Highest level of education		
Grade 12	13	19
Diploma/Certificate	33	49
Degree	22	32
Post-graduate degree	33	49

**Table 2:** Respondents’ mean score, factor loadings, *p*-values and Cronbach’s alpha (α).

Construct	Item Description	Mean Score	Factor Loading	*p*-Value	α
Mood	Positive mood	2.22	0.668	**	0.717
	Negative mood	1.20	0.671	**	
Familiarity	Small chocolates	1.38	0.644	**	0.745
	Large chocolates	1.73	0.571	**	
Acceptability	Acceptability	4.23	0.842	**	
Attitude	Negative	2.33	0.672	**	0.748
	Emotional	3.01	0.690	**	
	Functional	2.20	0.679	**	
	Obsession	2.78	0.514	**	
Sensory characteristics	Sensory sweetness	1.76	0.693	**	0.730
	Sensory bitterness	0.95	0.532	**	
Emotional response	Positive emotion	1.52	0.793	**	0.774
	Negative emotion	0.49	0.589	**	

Note. ** Statistically significant at the *p* ≤ 0.05 level.

**Table 3:** Correlations between consumer behaviour variables and emotional response of three chocolates.

	Chocolate 1 (Diabetic)			Chocolate 2 (Premium)			Chocolate 3 (Mainstream)		
	Positive Emotion	Negative Emotion	Guilty	Positive Emotion	Negative Emotion	Guilty	Positive Emotion	Negative Emotion	Guilty
Mood									
Positive mood	0.081	−0.038	0.066	0.114	−0.011	−0.054	0.245 **	−0.083	0.017
Negative mood	0.044	0.082	0.123	0.038	0.138	0.335 **	0.106	0.114	0.218 *
Familiarity									
Small chocolate format	0.041	0.029	0.091	0.209 *	−0.145	0.145	0.272 **	−0.119	0.075
Large chocolate format	0.135	0.032	0.028	0.232 **	−0.103	0.062	0.231 **	−0.095	0.058
Acceptability	0.463 **	−0.460 **	0.057	0.010	0.081	0.057	0.094	−0.057	−0.077
Attitudes									
Emotional Attitude	0.073	0.070	0.005	0.086	0.060	0.150	0.206 *	−0.018	0.103
Obsession Attitude	0.119	0.163	−0.020	0.132	0.091	0.050	0.214 *	−0.081	0.024
Negative Attitude	−0.199	0.036	0.230 **	−0.258	0.278	0.299 **	−0.092	0.136	0.309 **
Functional Attitude	0.047	0.007	−0.058	0.095	−0.001	0.099	0.117	−0.014	−0.062

Notes: Interpretation of Spearman’s rank order correlations based on Cohen’s guidelines (1977) (r): r = 0–r < 0.3 = Small, r ≥ 0.3–r < 0.5 = Moderate, r ≥ 0.5 = Strong; Tendencies to correlate (r ≥ 0.3) are indicated in bold; * Correlation is significant at the *p* ≤ 0.05 level (2-tailed), ** Correlation is significant at the *p* ≤ 0.01 level (2-tailed).

**Table 4:** Multiple regression analysis between emotion and sensory factors.

Dependent Variable	R^2^	R^2^ adj.	Independent Variable	
			Sensory Sweetness	Sensory Bitterness
			Beta (β) Coefficient	Beta (β) Coefficient
Chocolate 1 (Diabetic)				
Positive emotion	10.8%	9.5%	0.481 **	−0.183
Negative emotion	25.2%	24.1%	−0.378 **	0.330 **
Chocolate 2 (Premium)				
Positive emotion	7.7%	6.4%	0.132	0.427 **
Negative emotion	1.6%	0.0%	0.040	0.123
Chocolate 3 (Mainstream)				
Positive emotion	2.7%	1.3%	0.032	0.213
Negative emotion	3.5%	2.0%	0.098	0.061

Notes: R^2^ adj. = R^2^ adjusted; Beta (β) coefficient: β ≥ 0.2 = Small effect or tendency; β ≥ 0.5 = Mode-rate influence; β ≥ 0.4 = Practically significant; ** Relationship is significant at the *p* ≤ 0.01 level (2-tailed).

## Data Availability

The data presented in this study are available on request from the corresponding author.

## Appendix A

Questionnaire		
Section 1: Mood (0 = Not at all; 1 = Slightly; 2 = Moderately; 3 = Very; 4 = Extremely) Please complete the below questionnaire by ticking the appropriate box for each adjective, to give an indication of your mood at the moment. Please work quickly and do not think too long about the answer.		
Wide awake. Relaxed. Depressed. Friendly. Anxious. Clumsy. Cheerful. Drowsy. Aggressive. Clear-headed. Well-coordinated. Confused.		
Section 2: Familiarity (1 = Daily; 2 = Weekly; 3 = Monthly; 4 = Once a year/rarely; 5 = Never) Please indicate how often you consume the following types of chocolates.		
Segment Alfajores (Sandwich biscuit with chocolate centre or covered biscuits). Bagged selflines/softlines (Small identical items such as chocolate buttons). Standard boxed assortments (Bite size chocolates, often with fillings). Twist wrapped miniatures (Miniature versions of chocolate bars, wrapped). Chocolates with toys. Countlines (Chocolate bars). Seasonal chocolate. Filled tablets (slabs). (With a soft filling or hard pieces such as biscuits, nuts, mint). Plain dark tablets (slabs). Plain milk tablets (slabs). Plain white tablets (slabs).		Examples (Brand names not included due to ethical reasons)
Section 3: Acceptability (3 chocolate samples) Please indicate which below statement you agree with for each sample (select one). I would eat this chocolate every opportunity I had. I would eat this very often. I would frequently eat this. I like this and would eat it now and then. I would eat this if available but would not go out of my way. I do not like it but would eat it on occasion. I would hardly ever eat this. I would eat this only if there were no other food choices. I would eat this only if I were forced to.		
Section 4: Sensory characteristics (0 = Not at all; 1 = Slightly; 2 = Moderately; 3 = Very; 4 = Extremely) In the cooler bag you will find (three) samples of chocolate. Each sample is marked with a 3-digit code. Please ensure that the code on the sample matches the code on the survey when answering the questions. Please cleanse your palate before you start and in between each sample by drinking some of the water and eating one of the biscuits provided. Please taste sample xxx and then review below characteristics and definitions of the properties of the product you have just tasted. Please indicate how intensely you experienced each sensory property in the product.		
Descriptor	Definition	Reference
Appearance		N/A
1. Gloss	The amount of light reflected from the surface	
Aroma		Confectionary and sweets
2. Sweet	Aroma characteristic of sweet products	
Flavour		
3. Bitter	Taste of aloe, caffeine and hop bitters	Coffee, aloe juice, tonic water
4. Burnt	Flavour of overheated or scorched starch or sugar	Burnt sugar, burnt toast
5. Cocoa powder	Brown, sweet often bitter flavour	Cocoa beans, cocoa powder
6. Coffee	Flavour of coffee and coffee beans	Coffee power, coffee beans
7. Milky	Flavour associated with milk and milk products	Milk and cultured dairy products
8. Sweet	Taste delivered by sugar	Sucrose, fructose, artificial sweeteners
9. Cocoa powder	Brown, sweet often bitter flavour	Cocoa beans, cocoa powder
10. Vanilla	Flavour of vanilla	Vanilla pods, vanilla essence
Mouthfeel		N/A
Adherence	The amount that sticks to tooth surfaces	
11. Creaminess	Resembles the consistency of cream	N/A
Melting	The length of time for it to melt in the mouth	N/A
12. Mouthcoating	The amount of film or coating left on mouth surfaces	N/A
13. Milky	Flavour associated with milk and milk products	N/A
Aftertaste		
14. Bitter	Aftertaste delivered by substances such as quinine, caffeine and hop bitters	Coffee, aloe juice, tonic water
Section 5: Emotional response (0 = Not at all; 1 = Slightly; 2 = Moderately; 3 = Very; 4 = Extremely Please taste sample xxx and then review below characteristics of how the product makes you feel. Please rate how intensely you experience these emotions when eating the product.		
Active. Adventurous. Aggressive. Bored. Calm. Disgusted. Enthusiastic. Free. Good. Good-natured. Guilty. Happy. Interested. Joyful. Loving. Mild. Nostalgic. Pleasant. Satisfied. Secure. Tame. Understanding. Warm. Wild. Worried.		
Section 6: Attitude (1 = Strongly agree; 2 = Agree; 3 = Neither agree nor disagree; 4 = Disagree; 5 = Strongly disagree) Please indicate how much you agree or disagree with the below statements about chocolate in general.		
I eat chocolate to cheer me up when I am down. My desire for chocolate often seems overpowering. I feel unattractive after I have eaten chocolate. I often feel sick after eating chocolate. I eat chocolate as a reward when everything is going really well for me. I am often on one kind of diet or another. The thought of chocolate often distracts me from what I am doing (e.g., watching TV). I usually find myself wanting chocolate during the afternoon. I consider chocolate to be high in fat and of poor nutritional value. After eating chocolate I often wish I had not. I feel guilty after eating chocolate. I eat chocolate only when I am hungry. Chocolate often preys on my mind. I feel unhealthy after I have eaten chocolate. I always look at the kilojoule value of a chocolate snack before I eat it. If I resist the temptation to eat chocolate I feel more in control of my life. Nothing else but chocolate will satisfy my chocolate cravings. Even when I do not really want any more chocolate I will often carry on eating it. I eat chocolate to keep my energy levels up when I am doing physical exercise I eat more chocolate in the winter when it is colder. I often go into a shop for something else and end up buying chocolate. I feel depressed and dissatisfied with life after eating chocolate. I often eat chocolate when I am bored. I like to indulge in chocolate.		

## Appendix B

**Table A1:** Mean values of consumers’ acceptability and positive and negative emotions of three chocolate samples.

	Acceptability	Positive Emotions	Negative Emotions
Chocolate 1	4.8	1.46	0.65
Chocolate 2	4.5	1.37	0.60
Chocolate 3	3.4	1.92	0.25

Note. Acceptability scale: 1 = I would eat this chocolate every opportunity I had, 2 = I would eat this very often, 3 = I would frequently eat this, 4 = I like this and would eat it now and then, 5 = I would eat this if available but would not go out of my way, 6 = I do not like it but would eat it on occasion, 7 = I would hardly ever eat this, 8 = I would eat this only if there were no other food choices, 9 = I would eat this only if I were forced to; Emotions scale: 0 = Not at all, 1 = Slightly, 2 = Moderately, 3 = Very, 4 = Extremely; Interpretation of mean scores: <1.0 = Not present at all, ≥1.0–<2.0 = Slightly present, ≥2.0–< 3.0 = Moderately present, ≥3.0–<4.0 = Very present and 4.0 = Extremely present.
